# NOGO-B promotes EMT in lung fibrosis via MMP14 mediates free TGF-beta1 formation

**DOI:** 10.18632/oncotarget.20297

**Published:** 2017-08-16

**Authors:** Ye Xiong, Jing Zhang, Lingzhi Shi, Yunye Ning, Ying Zhu, Si Chen, Meng Yang, Jingyu Chen, Guo-Wu Zhou, Qiang Li

**Affiliations:** ^1^ Department of Respiratory Medicine, Changhai Hospital, Second Military Medical University, Shanghai, China; ^2^ Department of Pathology, Changhai Hospital, Second Military Medical University, Shanghai, China; ^3^ Department of Respiratory Medicine, Wuxi People Hospital, Nanjing Medical University, Nanjing, China; ^4^ Jiangsu Province Key Laboratory of Organ Transplantation, Wuxi People Hospital, Nanjing Medical University, Nanjing, China; ^5^ Department of Respiratory Medicine, Shanghai First Hospital, Shanghai Jiaotong University, Shanghai, China; ^6^ National Clinical Research Center for Respiratory Diseases, Center for Respiratory Diseases, Department of Pulmonary and Critical Care Medicine, China-Japan Friendship Hospital, Beijing, China

**Keywords:** Nogo-b, MMP14, idiopathic pulmonary fibrosis (IPF), epithelial mesenchymal transition (EMT), TGF-beta1

## Abstract

Idiopathic pulmonary fibrosis (IPF) is a lung disease with an extremely poor prognosis. Epithelial mesenchymal transition (EMT) appearing on the airway epithelial cell plays an essential role in the formation and development of Idiopathic pulmonary fibrosis. In this paper, Bleomycin (BLM)-induced mice model combined with bioinformatics analysis were employed to elucidate the potential mechanism of EMT in pulmonary fibrosis. The obtained results showed that endoplasmic reticulum protein Nogo-b may promote MMP14-mediated proprotein maturation of TGF-β1, accelerating the release of free TGF-β1 in type II airway epithelial cells A549, subsquently, induce the epithelial-mesenchymal transition (EMT) of the cell. In all, the overexpression of Nogo-b play a role in the course of pulmonary fibrosis by influencing the EMT ability of cells.

## INTRODUCTION

Losing control of wound healing process in response to tissue injury may lead to tissue overgrowth, fibrosis, and organ failure, one of the adverse consequences of fibrotic diseases is that it can affect many organs, especially for lungs [1–2]. Normal pulmonary fibrosis is pathologically featured with aberrant activation of epithelial mesenchymal transition (EMT) of airway epithelial cell accompanied by inflammation, fibroblast proliferation, and excessive collagen deposition [3–9]. EMT, as a leading factor for the pathogenesis of normal lung parenchyma replacement, is characterized by loss of epithelial characteristics (E-cadherin) and has acquired mesenchymal phenotype including N-cadherin, vimentin and smooth muscle actin (α-SMA) [10]. And also it was reported that the EMT process in lung fibrosis largely induced by the TGF-β1 via increasing the expression of the SNAI transcription factors [9], which is consistent with the recent reports on that EMT plays the key roles for pulmonary fibrosis [10–11]. Thus, it is of great significance to deeply understand the mechanisms of EMT of airway epithelial cell in pulmonary fibrosis. In addition, TGF-β1 induced disturbances of the homeostatic microenvironment resulted in Idiopathic pulmonary fibrosis (IPF) [12]. Idiopathic pulmonary fibrosis (IPF), as a kind of fibrotic diseases, has an extremely poor prognosis for patients, which is worse than that of numerous cancer patients in survival [13]. So far, in clinic, anti-inflammation drugs have been adopted as a long-term effective pharmaceutical treatment in a number of cases. Whereas, IPF suffers benefited few from these drugs. so it is urgent to develop new approaches to overcome this problem.

Nogo-b, a member of the endoplasmic reticulum protein family, is located on the endoplasmic reticulum and cellular membrane and abundant in peripheral tissues including lung [14]. It plays a vital role in repairing and regenerating the vascular and in the process of hepatic fibrosis [15–16]. Meanwhile, researches revealed that Nogo-b could promote cell migration in numerous tumors by intensifying EMT. In addition, our previous works showed that Nogo-b was essential for the migration and contraction of smooth muscle cells for airway and the acute lung injury induced by LPS [17]. However, there are only few researches on the performance of Nogo-b for pulmonary fibrosis and in the process of EMT concerning pulmonary fibrosis.

Matrix metalloproteinase 14 (MMP14) is a kind of membrane-type matrix metalloproteinases. It has a C-terminal transmembrane domain and a cytoplasmic tail [18]. Although MMP14 has been studied in a number of tumor cases and has long been considered it was correlated with EMT and featured with a capacity of enhancing cell invasion and migration [18–19], little is known about its significances in IPF [20–22].

In this study, we identified the effect of Nogo-b in EMT of and discussed its relationship with MMP14, which further clarify the effect of Nogo-b/MMP14 on EMT, as well as the role of EMT in the pathogenesis of IPF.

## RESULTS

### Ectopic expression of Nogo-b was observed in pulmonary fibrosis induced by bleomycin

**Figure 1:**
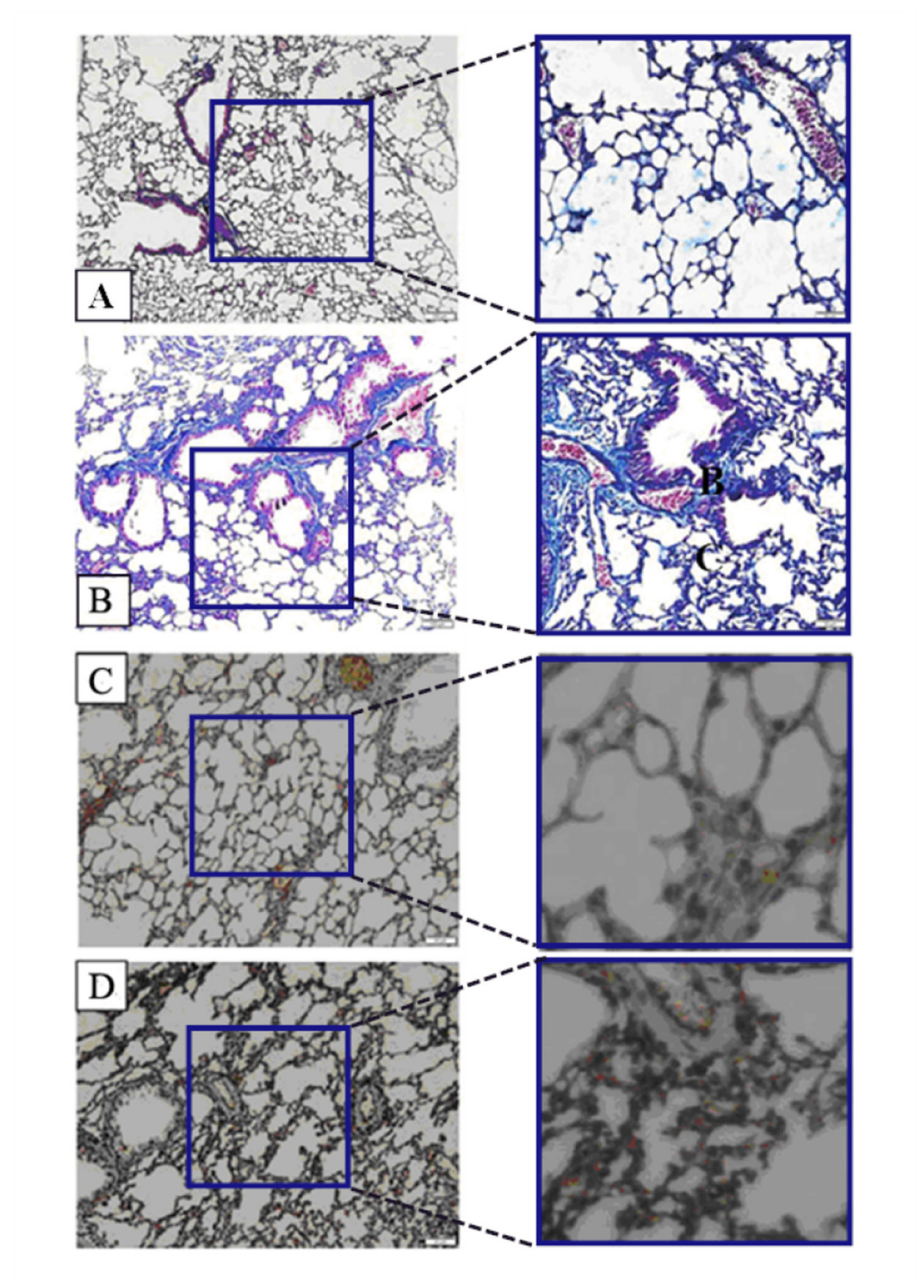
Nogo-b is up-regulated in a mouse model of bleomycin induced pulmonary fibrosis Representative images of Masson staining control lung tissue **(A)** and lung tissue in a model of BLM-induced pulmonary fibrosis **(B)**. Representative images of Nogo-b immunohistochemical staining control lung tissue **(C)** and lung tissue in a model of BLM-induced pulmonary fibrosis **(D)**.

First, Masson staining was used to identify the BLM-induced pulmonary fibrosis model in mice. As can be seen in Figure 1, Lung tissues in the control group are featured with continuous structure, intact wall of bronchial mucous membrane and normal lung alveoli. Smooth muscle cells surrounding the bronchial wall are red. Slight blue stain is seen around bronchus and vessels of all levels (Figure 1A). However, the BLM-based group revealed the increased alveolar wall. The structure of alveolar wall was damaged. Most of the area within the field of view presented bundles- or patches-shaped blue blocks (Figure 1B). It means huge deposition of collagen and fibronectin (one of the typical performances of pulmonary fibrosis, frequently accompanied by EMT). In order to determine whether protein expressions were associated with pulmonary fibrosis, Immunohistochemistry (IHC) was applied to examine the difference in the expression level of Nogo-b in lung samples of mice between the group treated with BLM and the untreated control, It was found that the lung mesenchyme in the mice treated with BLM showed a stronger brown staining for Nogo-b (Figure 1D) than that for the normal group (Figure 1C), indicating that high expression of Nogo-b is one of the features for the fibroblastic foci induced by BLM.

### Nogo-b can promote the EMT in type II alveolar epithelial cells

**Figure 2:**
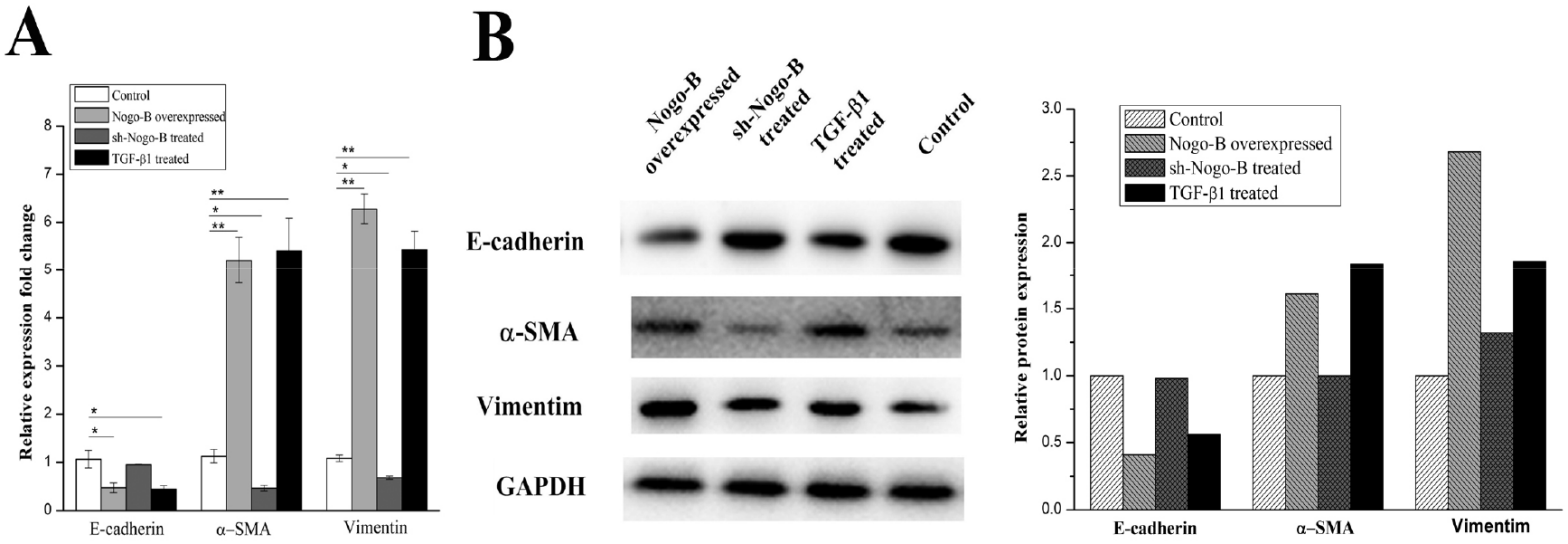
Overexpression of Nogo-B promotes EMT in A549 cell RT-PCR analysis **(A)** and Immunoblot analysis **(B)** of Marker proteins (E-cardherin, α-SMA and Vimentin) which were relative to the EMT in the A549 cells treated with Nogo-B overexpressed, sh-Nogo-B and additional TGF-β1 respectively. (The mRNA expression of Nogo-B in A549 cell lines was relative to the GAPDH mRNA from the same sample). All data are expressed as the means±SD of at least three.

In this section, we examined the effect of Nogo-b on EMT. Using TGF-β1 as a positive stimulator, we compared Nogo-b over-expression A549 cells and normal A549 cells in terms of the three major EMT markers: Vimentin, α-SMA and E-cadherin. For the sample treated with TGF-β1, its mRNA level of *Vimentin* and *α-SMA* was significantly increased (p < 0.05) in 48 hours, and the mRNA of *E-cadherin* decreased to almost the half of the control (Figure 2A). and the level of the protein expression of the Vimentin, α-SMA and E-cadherin showed the same trend with the mRNA. That is, the TGF-β1 stimulated A549 cell had nearly twice the level in Vimentin and α-SMA, almost half for the E-cadherin, compared to the control (Figure 2B). for the sh-Nogo-B treated sample, the Vimentin, α-SMA and E-cadherin were same with control in the level of both mRNA and protein. while for the the A549 cells transfected with pcDNA3.1-Nogo-b, the corresponding mRNA and protein for Vimentin, α-SMA and E-cadherin were similar to that of TGF-β1 treated samples. In conclusion, these data indicate that Nogo-b can promote the EMT in type II alveolar epithelial cells.

### Analysis of differentially expressed genes (DEGs)

**Figure 3:**
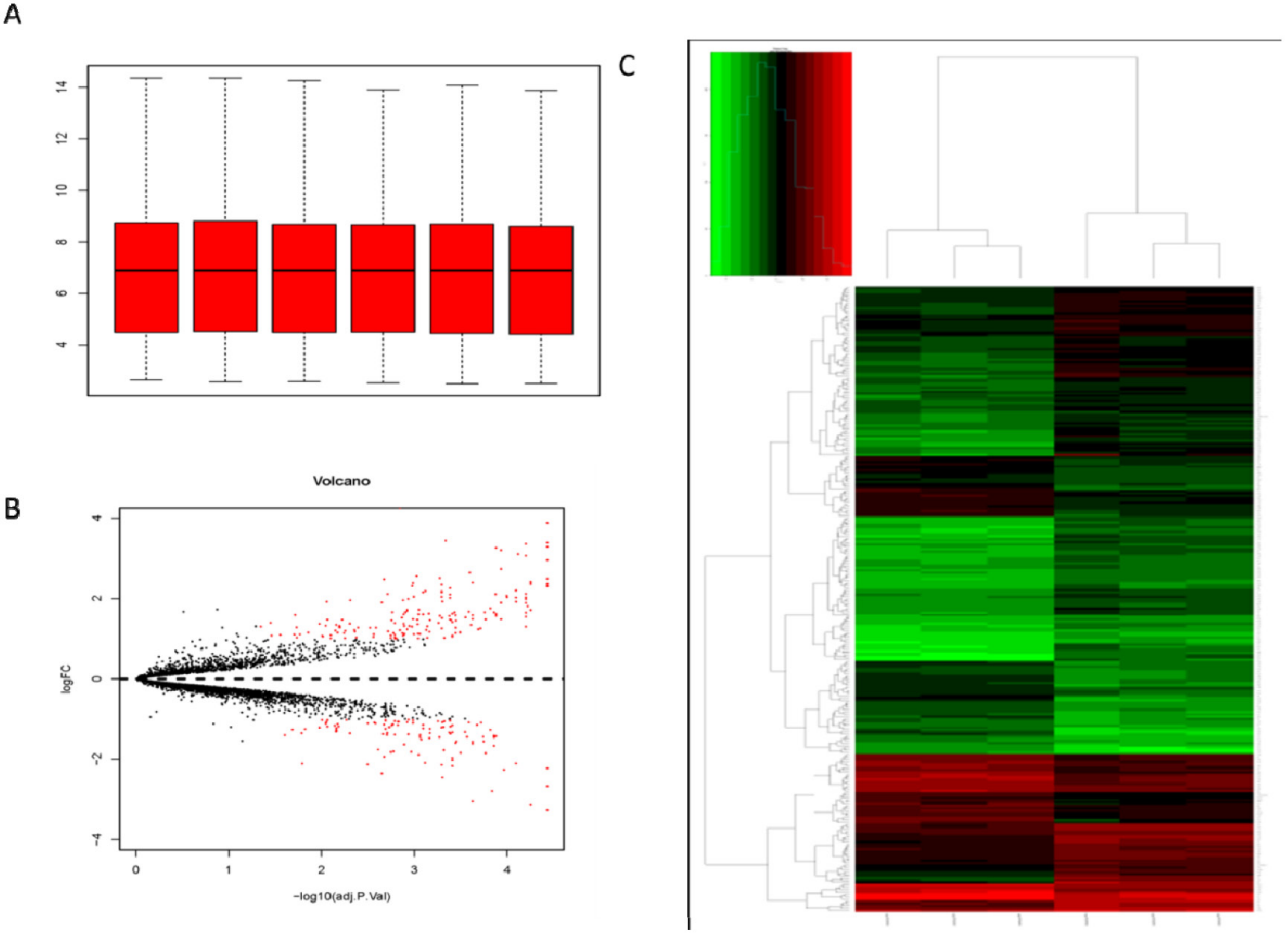
Analysis of DEGs by bioinformatics tools **(A)** Cassette figures of data distribution after standardization. The horizontal axis stands for sample names while the vertical axis represents the expression That all the black lines in the figure are almost on the same straight line reveals a good standardization degree. **(B)** The volcano plot of differentially expressed genes. The abscissa is logFC and the ordinates is -log10 (P Value). The red dots stand for the differentially expressed genes while the black dots represent genes not differentially expressed. **(C)** Hierarchical cluster dendrogram of DEGs. The horizontal axis represents sample names. GSM629991 GSM629992 and GSM629993 are saline treated wild type mice samples. GSM629994, GSM629995 and GSM629996 are Bleomycin treated wild type mice samples. The left vertical axis shows clusters of DEGs, and the above horizontal axis shows clusters of samples. Red represents up-regulated genes and green represents down-regulated genes.

The disease progress of pulmonary fibrosis is very complicated. A group of microarray data on the lung genes of mice induced by BLM were employed for the purpose of further studying the key genes affecting the occurrence and development of pulmonary fibrosis. The original data of GSE25640 was downloaded from GEO. Then the expression profile data was analyzed by Affy package in R language. Microarray data included a total of 5004 genes. Cassette figures after data standardization were shown in Figure 3A. All the black lines appearing in a straight line revealed a good standardization. By comparing the microarray data for the wild-type C57BL/6 mice treated with normal saline with that for the wild-type C57BL/6 mice treated with BLM, a total of 278 DEGs (accounting for 5.3% of the total genes) were discovered, including 172 up-regulated genes and 106 down-regulated ones (Figure 3B). Hierarchy cluster analysis indicated that the genes for three groups treated with Bleomycin distributed in a cluster and genes for another three groups treated with normal saline distributed in another cluster (Figure 3C). The results showed that the sequencing data was credible and may be directly applied to further analysis.

### Function and pathway enrichment analysis

**Figure 4:**
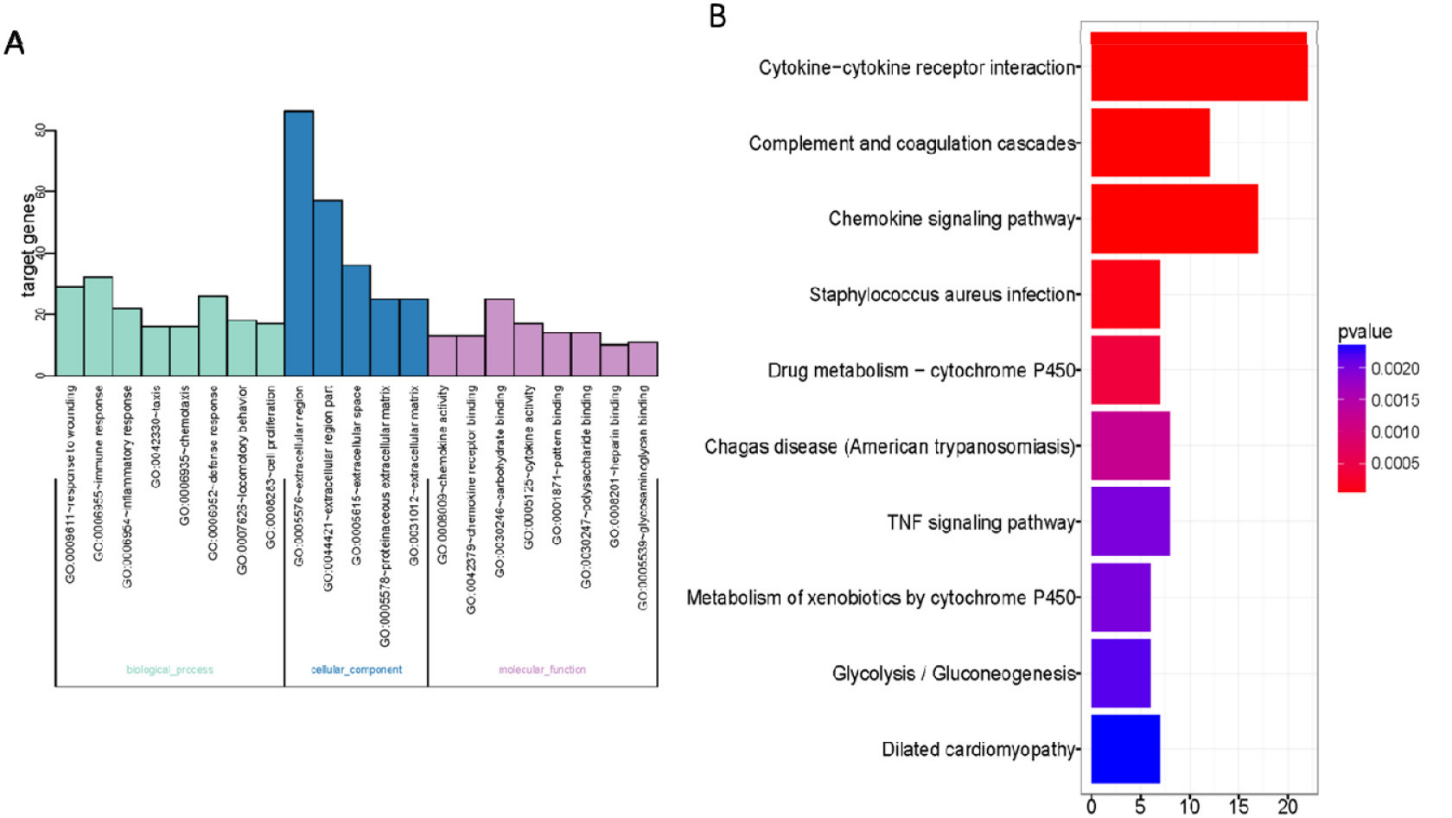
Gene Ontology enrichment analysis **(A)** Gene Ontology enrichment of DEGs of Bleomycin treated wild type mice lung samples; **(B)** KEGG enrichment of DEGs of Bleomycin treated wild type mice lung samples. The horizontal axis represents the number of enriched genes. The vertical axis represents the Gene Ontology or KEGG terms.

278 DEGs in gene list were uploaded to DAVID website. Based on the GO analysis, the enrichment pathways with the value of P less than or equal to 0.05 were obtained (Figure 4A). The GO pathways with highest enrichment were listed, from which we found that most of them were correlated with the extracellular region and extracellular region part. Top GO pathways with highest degree of enrichment were shown in Figure 4A.

We also carried out KEGG analyses on DAVID website (Figure 4B). Results revealed that interaction between cytokine and cytokine receptor was the KEGG pathway deserving our greatest attention. Among top 10 KEGG signal pathways and top 10 GO signal pathways with highest degree of enrichment, there were 17 flapped DEGs likely to be correlated to pulmonary fibrosis induced by BLM. The 17 flapped DEGs are: *SOCS3, Cc19, MMP14, Igf1, Aox3, C1qa, C1qb, C3ar1, Cc112, Cc12, Cc18, Cc19, Cxc110, Fcgr1, Gsta2, Kng1* and *Pdgfc*. Then, we tested changes in mRNA level using the method of RT-PCR based on the BLM-induced pulmonary fibrosis model that we built by ourselves. The results showed that differential expressions were existing in *MMP14*, *Ccl9*, *Socs3*, *Igf1*, *C1qa*, *C1qb*, *Ccl12*, *Ccl2*, *Ccl8*, *Cxcl10* and *Gsta2*. Verification results by RT-PCR are listed in Table 1. Among these candidate genes, MMP14 was selected as our target gene for further study, for it is considered to be the gene most likely to be correlated to pulmonary fibrosis and it has validated as the greatest differential expression genes in the list.

**Table 1:** Comparison of mRNAs by RT-PCR and RNA microarray

GENE	RT-PCR	Microarray	P-Value for RT-PCR	Gene Name
Socs3	2.47	2.07	<0.050	Suppressor of cytokine signaling 3
Ccl9	2.85	2.49	<0.050	Chemokine (C-C motif) ligand 9
Mmp14	3.4	2.31	<0.050	Matrix metalloproteinase-14
Igf1	2.33	2.32	<0.050	Insulin-like growth factor 1
C1qa	2.51	2.31	<0.050	complement C1q A chain
C1qb	2.33	2.37	<0.050	complement C1q B chain
Ccl12	2.1	2.64	<0.001	chemokine (C-C motif) ligand 12
Ccl2	1.99	2.11	<0.050	C-C motif chemokine ligand 2
Ccl8	2.88	3.36	<0.050	C-C motif chemokine ligand 8
Cxcl10	2.36	2.02	<0.001	C-X-C motif chemokine ligand 10
Gsta2	-2.53	-2.17	<0.001	glutathione S-transferase alpha 2

### Overexpression of MMP14 in type II alveolar epithelial cells generates a severe EMT phenotype

**Figure 5:**
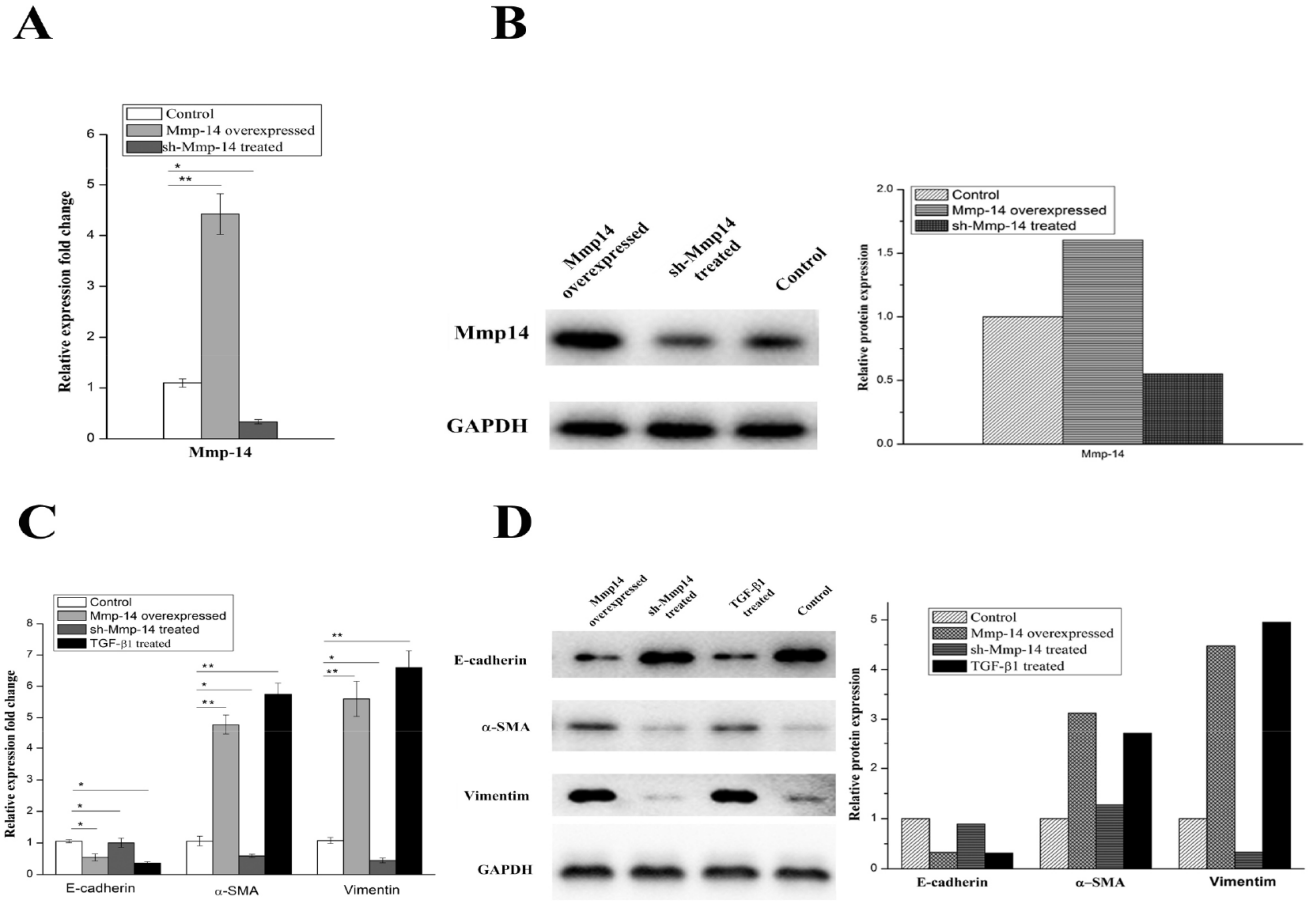
The protein and mRNA expression of Mmp14 in A549 treated with different ways **(A)** the mRNA expression of Mmp14 in A549 cell lines was relative to the GAPDH mRNA from the same sample. The experiments were performed three times, and the mean value±SD are presented. **(B)** Western blotting of Mmp14 in Blank, Mmp14 overexpressed and sh-Mmp14 treated A549. And quantitative analysis of Mmp14 expression in A549 cells under the indicated treatment Overexpression of Mmp14 promotes EMT in A549 cell. mRNA analysis **(C)** and Immunoblot analysis **(D)** of Marker proteins (E-cardherin, α-SMA and Vimentin) which were relative to the EMT in the A549 cells treated with Mmp14 overexpressed, sh-Mmp14 and additional TGF-β1 respectively. (The mRNA expression of Mmp14 in A549 cell lines was relative to the GAPDH mRNA from the same sample). All data are expressed as the means±SD of at least three independent experiments.

To confirm whether MMP14 would also affect the pulmonary fibrosis through EMT in human, we performed MMP14 over-expression and knockdown experiments in A549 cell. Over-expression and low-expression of MMP14 were verified by qRT-PCR and Western blot, as shown in Figure 5A and B. A significant up-regulation of MMP14 was observed when pcDNA3.1-MMP14 plasmid was transfected into A549 cell. And the down-regulation was observed when pLKO.1-shMMP14 plasmid was transfected into A549 cell.

Vimentin, α-SMA and E-cadherin are widely accepted as EMT markers. In subsequent experiments, it was found that TGF-β1, as an agonist of EMT, could induce a significant increase in level of agonist for vimentin and α-SMA (p < 0.01) (Figure 5C and 5D). Likewise, by comparing the A549 cell transfected by MMP14 for 48 hours with the negative control, it was found that its expressions of mRNA for Vimentin were also increased significantly (p < 0.01) (Figure 5C). Meanwhile, protein of Vimentin would witness an increased as the rising mRNA level (Figure 5D). In our researches, the over-expression of MMP14 would induce the significant increase of expressions of mRNA and protein for α-SMA (Figure 5C and 5D) (p < 0.01) as well. E-cadherin mRNA was remarkably decreased 48 hours after MMP14 was transfected, accompanied by the declining of E-cadherin protein level (p < 0.01) (Figure 5C and 5D). 48 hours after the treatment of A549 cell by TGF-β1, expressions of E-cadherin mRNA and protein were reduced. These results showed that MMP14 can stimulate A549 cell to manifest EMT features like TGF-β1.

### *Nogo-b* is identified as upstream controlling gene of *MMP14*

**Figure 6:**
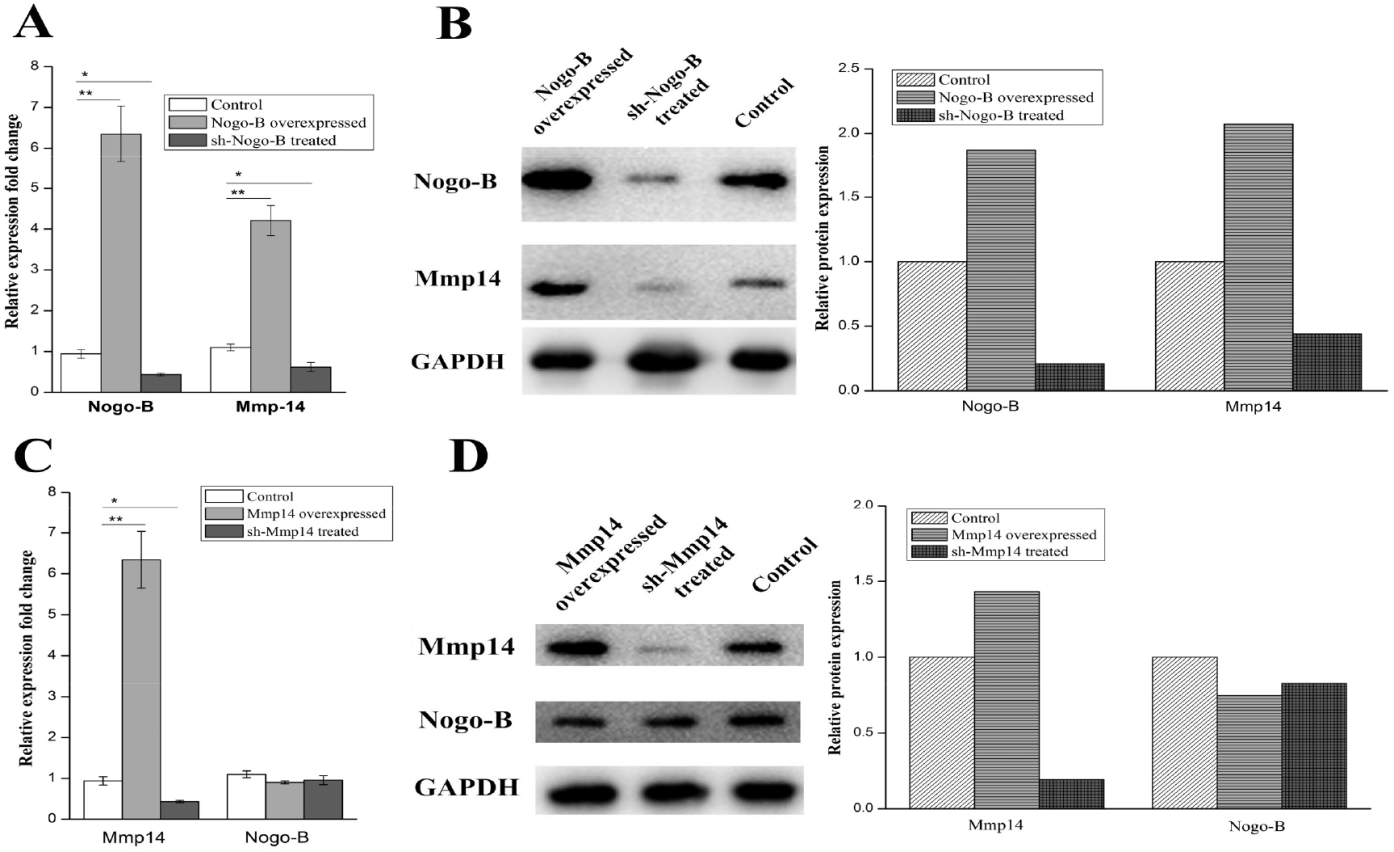
The protein and mRNA expression of the Mmp14 were in line with the up and down regulated Nogo-B in A549 cells **(A)** qRT-PCR analysis of Mmp14 and Nogo-B levels in the A549 cells with indicated treatments. All data are expressed as the means±SD of at least three independent experiments. The protein and mRNA expression of the Mmp14 were influenced by the up and down regulated Nogo-b in A549 cells. **(B)** Western blot analysis of Mmp14 in A549 cells treated with overexpresion and silence of Nogo-B, GAPDH was used as a loading control. **(C)** qRT-PCR analysis of Mmp14 and Nogo-B levels in the A549 cells with indicated treatments. All data are expressed as the means±SD of at least three independent experiments. **(D)** Western blot analysis of Nogo-B in A549 cells treated with overexpresion and silence of Mmp14, GAPDH was used as a loading control.

In order to demonstrate the molecular mechanisms by which MMP14 influenced EMT in type II alveolar epithelial cells, a panel of molecules was predicted to be potential targets of the MMP14. Our team had proved the key function of Nogo-b in migration and contraction of airway smooth muscle cells and lipopolysaccharide-induced acute lung injury [15–16]. Researchers also reported that Nogo-b is able to promote EMT in HeLa cervical cancer cells via Fibulin-5 [23]. Is there any correlation between MMP14 and Nogo-b? In this study, Expression difference of the MMP14 between Nogo-b overexpression A549 cell line and normal A549 cells was further examined. Firstly, we examined the expression quantity of MMP14 in A549 cells containing over-expression of Nogo-b, results of which showed that MMP14 was higher in the A549 cells containing high-expression of Nogo-b than that in cell line containing low-expression of Nogo-b (Figure 6A and 6B). Such result promoted us to further study whether the expression of Nogo-b is affected by the abnormal expression of MMP14. It was found that A549 cells tranfected by lipo2000/pcDNA3.1-MMP-14 plasmids seldom changed in expressions of mRNA and protein of Nogo-b (Figure 6A and 6B). Moreover, knockout of Nogo-b using sh-RNA significantly reduced the expression of MMP14. However, remarkable expression differences of Nogo-b did not appear in A549 cells of MMP14 knockout (Figure 6C and 6D). Therefore, we can draw a conclusion that expression of MMP14 is regulated and controlled by Nogo-b.

### Up-regulation of *MMP14* rescues Nogo-b mediated EMT

**Figure 7:**
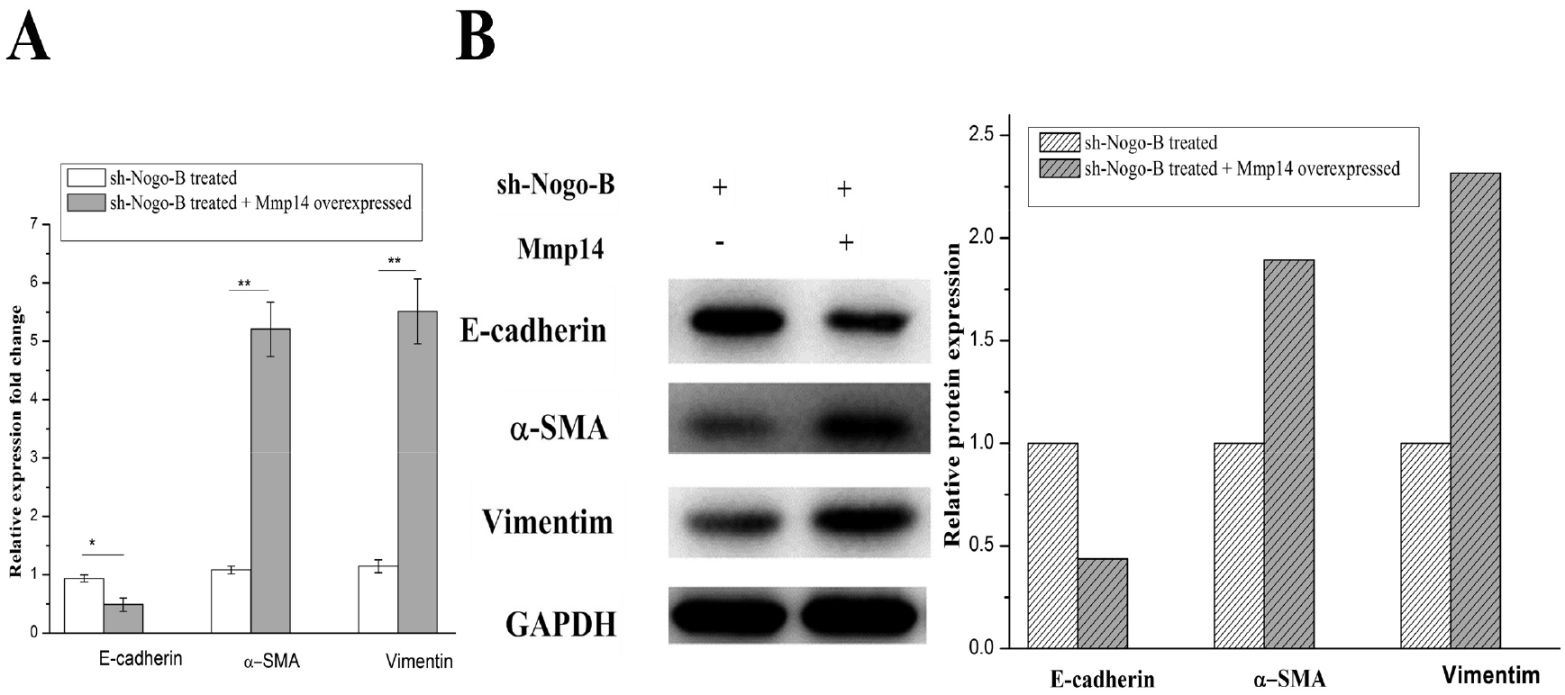
The EMT of the A549 cells with down-regulated Nogo-B were recovered by the up-regulated Mmp14 mRNA analysis **(A)** and immunoblot analysis **(B)** of marker proteins (E-cardherin, α-SMA and Vimentin) which were relative to the EMT in the A549 cells treated with Mmp14 overexpressed and Nogo-b knock down.

It was found that overexpression of MMP14, which is an upstream controlling gene of Nogo-b, could enhance EMT, based on which we hypothesized that Nogo-b could change the EMT of A549 by affect functions of MMP14. Silent A549 cells and normal A549 cells in Nogo-b were used as the experimental materials. As expected, shRNA which knocked down the Nogo-b remarkably reduced the EMT capabilities of A549 cells (Figure 7A and 7B). To further study EMT mechanism mediated by Nogo-b and MMP14 in type II alveolar epithelial cell model, we over-expressed MMP14 in silent A549 cells of Nogo-b. Results of which revealed that EMT capacities of cells would be intensified in cases where MMP14 was over expressed in A549 cells which knocked down by Nogo-b (Figure 7A and 7B). These results further verified that Nogo-b could affect the EMT capacity of A549 cells by regulating MMP14.

### MMP14 can promote TGF-β1 release from pro-proteins

**Figure 8:**
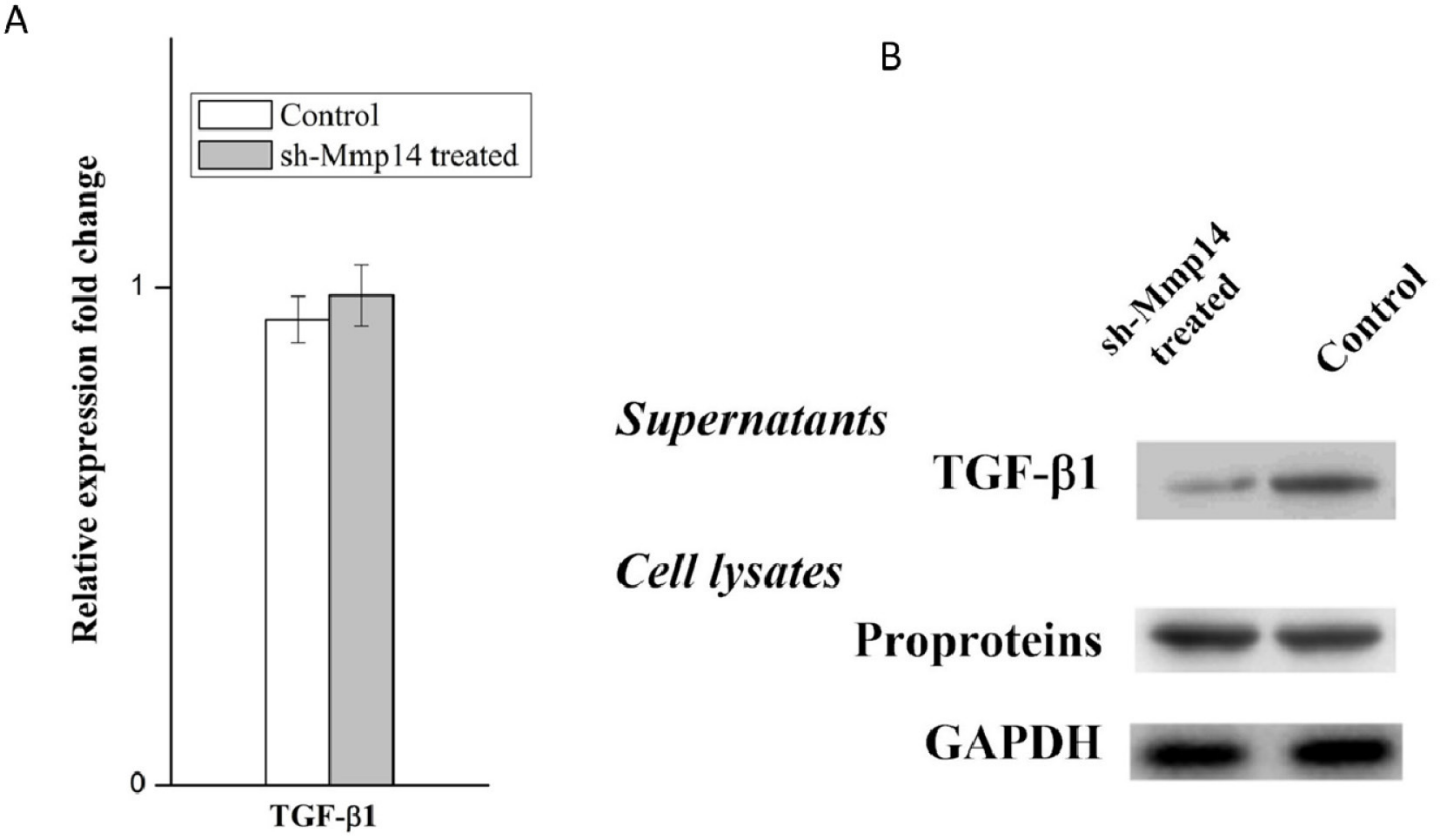
MMP14 promote TGF-β1 release from pro-proteins **(A)** TGF-β mRNA expression was quantified in the MMP14 knock down A549 cell model. **(B)** Free TGF-β1 in supernatants and TGF-ß proproteins in cell lysates were determined by Western blot.

**Figure 9:**
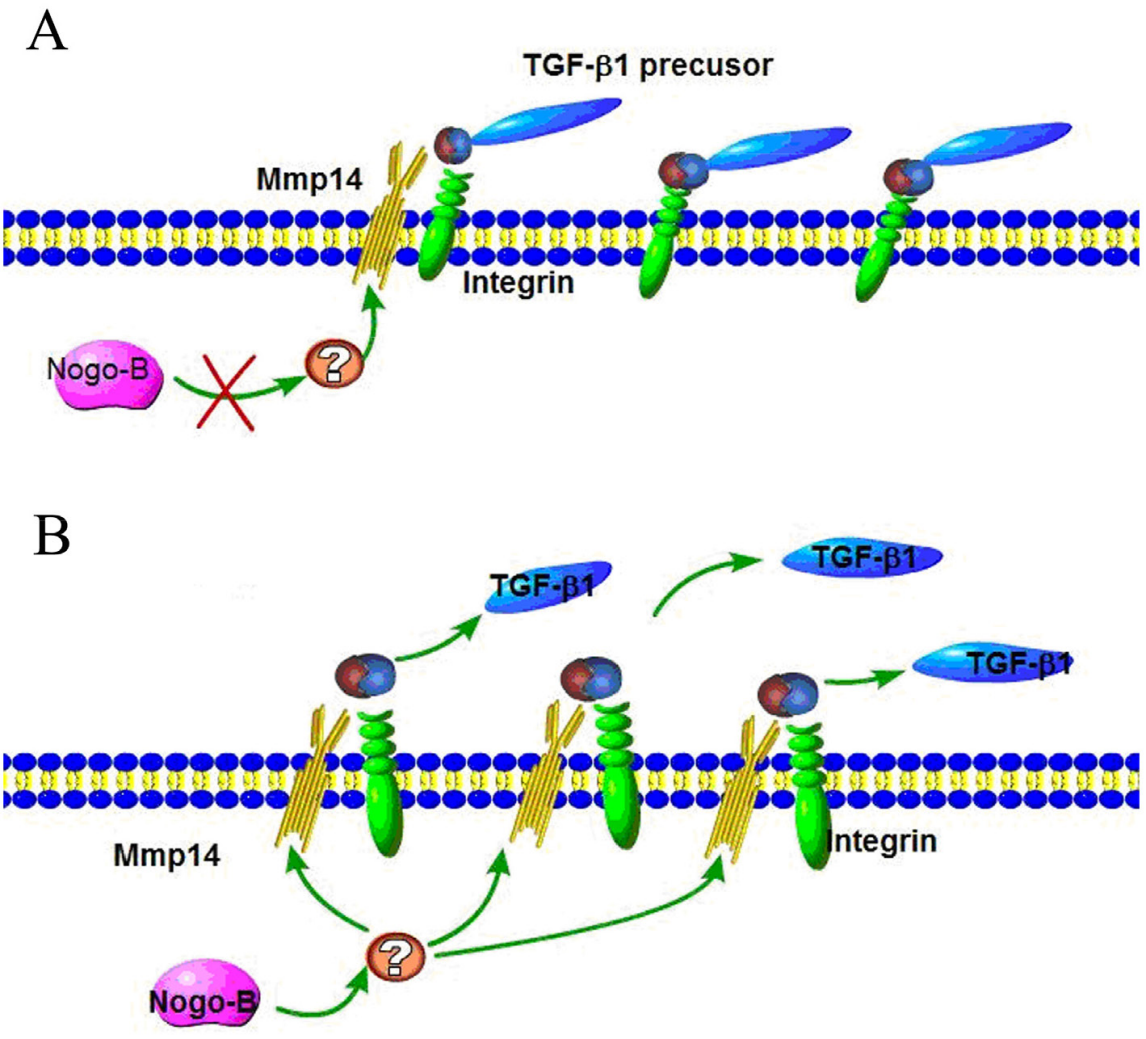
Activation of latent TGF by Nogo-b/MMP14 pathway **(A)** The integrin binds RGD site of latent TGF which can be proteolytically digest by MMP14; **(B)** Nogo-b increased MMP14 promoted the proteolytically digest of latent TGF, permitting more active TGFβ1 distant from receptors.

TGF-β1 is widely known as the main cytokine involved in the pathogenesis of IPF, which can induce EMT on the model of A549 type II airway epithelial cells. Anti-TGF-ß therapy is commonly used to cure fibrosis diseases. TGF-β family are translated as proproteins which can be activated and hydrolyzed by a number of proteases including MMP14. In this study, we hypothesized that the regulation of MMP14 by NOGO-B was performed by catalyzing the hydrolysis of TGF-β1 pro-proteins and releasing TGF-β1. To prove this point, we tested the expression quantity of TGF-β mRNA in the silent model of MMP14, results of which showed that there were no obvious differences between the silent model and non-silent model in expression of TGF-β mRNA (Figure 8A). Meanwhile, we found that less free TGF-β1 were observed in knockdown model of MMP14 while expression of TGF-β proproteins in cell lysate was not significantly changed (Figure 8B). These results suggested that MMP14 cleaved TGF-β proproteins in cell culture supernatants (Figure 9), which may be a molecular mechanism that Nogo-b/MMP14 pathway utilized to promote EMT of alveolar epithelial cells dependent by TGF-β1.

## DISCUSSION

Our study demonstrated that MMP14 played a critical role in EMT of pulmonary fibrosis, and the expression level of MMP14 determined the progress of EMT. Meanwhile, we discovered that Nogo-b could regulate the expression of MMP14. The absence of Nogo-b inhibited the expression of MMP14 while the absence of MMP14 would not affect the expression of Nogo-b. Moreover, it was identified that, Nogo-b could promote the EMT of A549 cell, and loss of MMP14 decreased the release of active TGF-ß1 but without any influences on the expression of TGFß proteinogen. In all, the obtained results demonstrated that MMP14 as a new downstream target of Nogo-b could mediate the promote pulmonary fibrosis, in addition, Nogo-b/MMP14 could regulate pulmonary fibrosis by affecting the process of cleaving TGFß proteinogens.

At present, although we have known that Nogo-b plays an important role in vascular injury repair, tissue repair and inflammation process [24–25], little is known about its functions in the lung. Previous works have shown that Nogo-b is irreplaceable for hepatic fibrosis [15], so it attract interestings from us to speculate that the functions of Nogo-b maybe related to the pulmonary fibrosis. Meanwhile, we have known the fact that Nogo-b is critical for the migration and contraction of airway smooth muscle cells as well as LPS-induced acute lung injuries [17, 26], LPS-induced acute lung injuries are frequently accompanied by pulmonary fibrosis, and pulmonary fibrosis is also associated with the reconstruction of airway smooth muscles. In this study, whether Nogo-b had related functions for the process of pulmonary fibrosis were verified.

BLM is an antineoplastic drug which can induce pulmonary fibrosis [27]. Exposure of BLM can lead to fatal pulmonary toxicity, which is the most commonly used to set up IPF model of mice to study disease pathogenesis with the characteristics of the patchy parenchymal inflammation, the reactive epithelial hyperplasia and the epithelial-mesenchymal transition [16, 28–31]. In this study, it was firstly observed that high expression of Nogo-b in lung tissues of BLM-induced pulmonary fibrosis models of mice, indicated that Nogo-b is associated with the occurrence of pulmonary fibrosis diseases. Meanwhile, the injured airway epithelial cell became the fibrocyte through EMT and played important roles in the occurrence and development of IPF. As a result, we examined relationship between Nogo-b and EMT based on A549 cell models of Type II airway epithelial cells. Results showed that the increase of Nogo-b expression can enhance EMT of the cell.

The pathogenetic process of pulmonary fibrosis is very complicated, which is accompanied by accumulation of a great many extracellular matrix and abnormal expression of inflammatory factors. In this study, the results derived from GO and KEGG analyses on the model of a group of BLM-induced mice in GEO showed a number of DEGs were abundant in pathways such as interactions of inflammatory factors and extracellular matrix by the comparison of the BLM-induced model and the control model. Subsequently, we found that MMP14 abundant in extracellular matrix and TNF signaling pathway was significantly increased in the pulmonary fibrosis model induced by bleomycin via RT-PCR verification. MMP14, a kind of matrix metalloproteinase, is regarded as a major effect factor for the degradation of extracellular matrix and core matrisome proteins [31–32]. In general, the MMP14 shows low activity under normal conditions [33]. While during the course of repairing and reconstruction, its expression is increased and leading to high corresponding activity [33–34]. A great number of researches have shown that MMP family expresses at a high level in complicated fibrosis diseases including IPF [35–37]. However, the problem that whether MMP14 plays an important role in IPF has never been discussed before.

It has been reported by many researchers that MMP14 has played a crucial role in tumor growth and migration process of EMT dependence [38]. In addition, MMP14 was known as Dexamethasone-dependent biomarker of EMT in primary hepatocytes [31]. In this article, we discovered that the over-expression of MMP14 in A549 cells could intensify EMT of cells, similar to the role of positive control of TGF-β1. However, cells of *MMP14* knock down were decreased in EMT capacities. These results revealed that MMP14 could enhance the EMT activity of cells and consequently resulted in the occurrence and development of pulmonary fibrosis. Moreover, we discussed the correlation between Nogo-b and MMP14, both of which were molecules that could promote EMT and cause pulmonary fibrosis. Results showed that Nogo-b was a upstream controlling factor of MMP14. The over-expression of Nogo-b could increase the expression of MMP14 as well as intensify the EMT of cells. While the increase or reduction of MMP14 would not exert any influence on expression of Nogo-b. In addition, we also found that the increase of MMP14 could recover the weakening of EMT arising from the silence of Nogo-b and the reduction of MMP14 could weaken the strengthening of EMT caused by Nogo-b. Therefore, we could draw a conclusion that Nogo-b relied on MMP14 to intensify the EMT for airway epithelial cells.

TGF-β1, which can enhance EMT of numerous cells, plays a key role in the process of pulmonary fibrosis [1, 12, 39]. Previous researches revealed that Nogo-b could cause hepatic fibrosis through TGFβ1/Smad2 signaling pathway [40]. Likewise, it was reported that MMP14 could increase activity of TGFβ1/Smad2 signaling pathway and raise stem cells in injured artery blood vessels to repair damages [41]. Furthermore, literatures previously showed that MMP14 could perform enzyme cleavage on TGFβ precursor proteins to release free TGFβ1 [42–43]. In this article, we discovered that MMP14 was controlled and regulated by Nogo-b and was correlated to pulmonary fibrosis. Hence, we hypothesized that Nogo-b/MMP14 signaling pathway was affecting the process of pulmonary fibrosis by influencing the TGFβ1 signaling pathway. We also proved our hypothesis. Results showed that reduction of MMP14 expression would neither affect the mRNA expression of TGF-β1 nor the expression of TGFβ precursor proteins. Instead, it would decline the release of free TGF-β1. All these results indicated that Nogo-b/MMP14 signaling pathway actually relied on the enhancement of enzyme cleavage of TGFβ precursor proteins rather than the influences on mRNA expression to affect the EMT of airway epithelial cell and pulmonary fibrosis.

In conclusion, MMP14/Nogo-b pathway serves as a new driver factor of the EMT process in BLM-induced pulmonary fibrosis, which can intensify EMT of cells by stimulating release of free TGF-β1. These findings can help us deeply understand the pathogenesis of IPF. In addition, the therapeutic schedule specific to MMP14/Nogo-b may effectively relieve the symptoms of pulmonary fibrosis, which is the task our team plans to work on.

## MATERIALS AND METHODS

### Establishment of a mouse model of BLM-induced pulmonary fibrosis

A total of 35 8-week-old male C57BL/6 mice were purchased from the Slaccas Experimental Animal Company (Shanghai, China). All animal experiments were carried out with approval from the Ethics Committee of Changhai Hosiptial. The mice were divided into the following 2 groups: i) The control group, ii) The group treated with BLM. Way of treatment with BLM (HisunPfizerPharmaceuticals Co., Ltd., Zhejiang, China) is as follows: 8-week-old mice were anesthetized with isoflurane. BLM (10 mg/kg body weight in 50 μl saline) was delivered into the oropharyngeal cavity after the tongues of the anesthetized mice were gently pulled forward. Tongues were kept pulled forward until intratracheal injection was completed. The mice in the control group without BLM treatment were injected with saline as the above steps. The mice were killed after 28 days, lung tissues of which were used for subsequent analysis.

### Masson staining

After mice were sacrificed, lungs were harvested, embedded in paraffin and then deparaffinized, followed by Masson staining for the observation of lung fibrosis.

### Immunohistochemistry (IHC)

Lung samples were fixed in formalin solution and embedded by paraffin. Paraffin blocks were cut into4μm-thickness sections and then incubated in the 3% hydrogen peroxide solution to eliminate endogenous peroxidase activities. Antigen retrieval was carried out by heat mediationin method in citrate buffer solution (pH= 6). Sections were closed in the 10% goat serum solution before being incubated in primary antibody containing Nogo-b (Abcam) overnight at a temperature of 4°C. PBS without primary antibodies was applied as the negative control. Immunohistochemical staining was performed with the 3-3’-diaminobenzidine [44] and then counterstain was followed by hematoxylin. The immunohistochemistry results were graded and classified into two groups according to the average staining intensity and area.

### Data analysis

The microarray data GSE25640, downloaded from Gene Expression Omnibus (GEO) database, was sequencing upon the platform of Affymetrix Mouse Genome 430 2.0 Array. This dataset was stored by Turashvili et al [20], It has 12 gene microarray data, including those for lungs of 6 wild type mice and 6 FIZZZ2 knockout mice. We picked out information for 6 wild type samples, among which three mice were treated with bleomycin and other three were not. The original CEL data was imported into R program and processed by affy package to correct and normalize the background. Moreover, multiple probe data used to inspect the expression of the same gene was summarized. Different Expression Genes (DEGs) between the bleomycin- treated group and the bleomycin-untreated group were identified using Limma package [22]. The p-value no more than 0.05 and |log2fold change| larger than 1 were regarded as the screen threshold value of the DEG.

### Cell culture and overexpression

The Type II alveolar epithelial cells A549 were cultivated in the DMEM culture medium containing 10% of fetal bovine serum (Gibco, USA). Cells were placed in a standard humidity incubator with a temperature of 37°C and containing 5% of CO_2_. TGF-β1(Sigma-Aldrich, USA) was added and the final concentration of the culture medium is 10 ng/ml. The cells were cultured in the medium containing TGF-β1 for 48 hours before being collected for further analysis.

A total of 1ug of pcDNA-3.1-MMP14, pcDNA-3.1-Nogo-b plasmid was diluted in 50 μl of RPMI 1640 medium free of serum and 2 μl of Lipofectamine®2000 was diluted in 50 μl of RPMI 1640 medium free of serum. Then mix the above two solutions and add the mixture into the A549 cell contained in the 24-well plate for transfection. After incubation at 37°C for 4 hours, the serum-free medium was substituted by a complete medium containing 10% of serum. Culture it for another 48 hours. The cells were then collected for subsequent experiments. After transfection for 48 hours, inspect expressions of mRNA and protein based on the technologies of real-time PCR and Western blotting.

### Nogo-b and MMP14 knock down

pLKO.1 plasmid was digested with EcoRI and AgeI. The short hairpin RNA (shRNA) carrier was constructed to target Nogo-b gene by inserting the sequence of 5’-CCGGGCAGTGTTGATGTGGGTATTTCTCGAGAAATACCCACATCAACACTGCTTTTTTG into the plasmid pLKO.1. The targeting of MMP14 plasmid was set up by inserting the sequence of 5’-CCGGGTGGATGGACACGGAGAATTTCTCGAGAAATTCTCCGTGTCCATCCACTTTTTG. Plasmids were then transfected into A549 cell. Stable cell lines of Nogo-b with low expression were screened and obtained by puromycin.

### Real-time PCR

Real-time PCR was analyzed by LightCycler® 480 Real-Time PCR system (Roche, Switzerland) with reagent of SYBR PrimeScriptTM RT Reagent Kit (TaKaRa, Japan). All the operation should follow the manufacturer’s operation manual. The primers and reaction conditions are shown in Table 2.

**Table 2:** RT-PCR primers

		Mus musculus	Homo sapiens
SOCS3	sense	5′-ATCCTGGTGACAT GCTCCTC-3′	
	anti-sense	5′-CAAATGTTGCTTCCCCCTTA-3′	
Ccl9	sense	5′-ATGAAGCCTTTTCATACTGCCCTC-3′	
	anti-sense	5′- TTATTGTTTGTAGGTCCGTG GTTG-3′	
Mmp14	sense	5′- GCTCC GAGGAGAGATGTTTG-3′	5′- GGAGACAAGCATTGGGTGTT-3′
	anti-sense	5′-CATCACTGCCCATG AATGAC-3′	5′-GGTAGCCCGGTTCTACCTTC-3′
Igf1	sense	5′-ACTGACATGCCCAAGACTCAGAAGTC-3′	
	anti-sense	5′-TGCCTCCGTTACCTCCTCCTGTTC-3′	
C1qa	sense	5′-ACAAGGTCCTCACCAACCAG-3′	
	anti-sense	5′-AAGATGCTGTCGGCTTCAGT-3′	
C1qb	sense	5′-AGTGCCGGGCCTCTACTACT-3′	
	anti-sense	5′-CGGGAAACAGTAGGAAACCA-3′	
Ccl12	sense	5′-ATCAGTCCTCAGGTATTGGCTGGA-3′	
	anti-sense	5′-TGGCTGCTTGTGATTCTCCTGTAG-3′	
Ccl2	sense	5′-TTAAAAACCTGGATCGGAACCAA-3′	
	anti-sense	5′-GCATTAGCTTCAGATTTACGGGT-3′	
Ccl8	sense	5′-AGCCCAGGCACCATCTGCTTGTAA-3′	
	anti-sense	5′-TGCCCCATGGAAGCTGTGGTTTTC-3′	
Cxcl10	sense	5′-TCAGCACCATGAACCCAAG-3′	
	anti-sense	5′-CTATGGCCCTCATTCTCACTG-3′	
Gsta2	sense	5′-TTATGTCCCCCAGACCAAAG-3′	
	anti-sense	5′-CCTGTTGCCCACAAGGTAGT-3′	
Gapdh	sense	5′-CCTTCATTGACCTCAACTAC-3′	
	anti-sense	5′-TGGGCCCTCAGATGCCTGCT-3′	
RTN4	sense		5′-GGAGCTGCAAAGCAGATCGTGAC-3′
	anti-sense		5′-GGCTGGCACCAAACACCACTCC-3′

### Western blotting analysis

The protein concentration was determined using the Bio-Rad protein assay system. Cells receiving different treatment were dissolved in Laemmli buffer and boiling for 5 minutes. 20ug of protein was subjected to electrophoresis in 12% of SDS-PAGE and then transferred to nitrocellulose membrane. Keep it closed in PBS containing 5% skimmed milk for 2 hours at room temperature and then add different primary antibodies including Nogo-b (Abcam, dulited in PBS with 1:1000); MMP14 (Santa Cruz, dulited in PBS with 1:200); E-cadherin; vimentin or α-SMA both from ImmunoWay Biotechnology Company, dulited in PBS with 1:1000; GAPDH was regarded as the internal refere nce, dulited in PBS with 1:1000. After washing, the membranes were incubated in the corresponding secondary antibody for 1 hour at room temperature, followed by chemiluminescence testing strips.

### Statistical analysis

All data are presented as the means ± standarddeviation (SD). Comparisons among groups wereperformed by one-way ANOVA using SPSS 17.0 software (SPSS, Inc., Chicago, IL, USA). All experiments were repeatedthree times. P<0.05 was considered to indicate a statistically significant difference.

## Abbreviations:

Nogo-b: reticulon 4-b
MMP14: matrix metallopeptidase 14
IPF: Idiopathic pulmonary fibrosis
EMT: Epithelial mesenchymal transition
TGFbeta1: transforming growth factorbeta 1
